# Electroclinical characteristics of seizures arising from the precuneus based on stereoelectroencephalography (SEEG)

**DOI:** 10.1186/s12883-018-1119-z

**Published:** 2018-08-13

**Authors:** Yanfeng Yang, Haixiang Wang, Wenjing Zhou, Tianyi Qian, Wei Sun, Guoguang Zhao

**Affiliations:** 1 Department of Neurology, Xuan Wu Hospital, Capital Medical University, No 45, Changchun Street, Xicheng District, Beijing, 100053 China; 2 Department of Neurosurgery, Xuan Wu Hospital, Capital Medical University, No 45, Changchun Street, Xicheng District, Beijing, 100053 China; 3 Epilepsy Center, Yuquan hospital, Tsinghua University, Beijing, 100049 China; 4 Department of Radiology, Beijing Key Lab of magnetic resonance imaging (MRI) and Brain Informatics, Xuan Wu Hospital, Capital Medical University, Beijing, 100053 China

**Keywords:** Precuneal epilepsy, SEEG, Electroclinical characteristics, Anatomical-electrical-clinical correlations

## Abstract

**Background:**

Seizures arising from the precuneus are rare, and few studies have aimed at characterizing the clinical presentation of such seizures within the anatomic context of the frontoparietal circuits. We aimed to characterize the electrophysiological properties and clinical features of seizures arising from the precuneus based on data from stereoelectroencephalography (SEEG).

**Methods:**

The present retrospective study included 10 patients with medically intractable epilepsy, all of whom were diagnosed with precuneal epilepsy via stereoelectroencephalography (SEEG) at Yuquan Hospital and Xuan Wu Hospital between 2014 and 2016. Clinical semiology, scalp electroencephalography (EEG) findings, magnetic resonance images (MRI), and positron emission tomography (PET) images were analyzed during phase I preoperative evaluations. Following electrode implantation, the semiological sequence, ictal SEEG evolution, and anatomy of the relevant brain structures were analyzed for each seizure.

**Results:**

Seven of ten patients reported auras, including body image disturbance (2/7), vestibular responses (2/7), somatosensory auras (1/7), visual auras (1/7), and non-specific auras (1/7). Primary motor manifestations included bilateral asymmetric tonic seizures (BATS) (7/10) and hypermotor seizures (HMS) (3/10). In one patient, epileptiform discharge on interictal EEG occurred ipsilateral to the side of the epileptogenic zone (EZ). Discharge was non-lateralized in the remaining nine patients. In six patients, interictal EEG signals were primarily localized in the temporal–parietal–occipital area. In two patients, ictal onset occurred ipsilateral to the EZ, which was mainly located in the temporal–parietal–occipital area. Two patterns of seizure spread were observed. The first pattern was characterized by BATS activity with ictal spread to the supplementary motor area (SMA), paracentral lobule (PCL), precentral gyrus (PrCG), or postcentral gyrus (PoCG). The second pattern was characterized by HMS activity with ictal spread to middle cingulate cortex (MCC) and posterior cingulate cortex (PCC).

**Conclusion:**

Aura type (e.g., body image disturbance and vestibular response), BATS, and HMS are the main indicators of precuneal epilepsy. Scalp EEG is of little use when attempting to localize precuneal seizures. Our findings indicate that the clinical characteristics of precuneal epilepsy vary among patients, and that the final electro–clinical phenotype depends on the pattern of seizure spread.

## Background

The precuneus is a discrete area located in the posterior region of the medial parietal cortex, neighbored anteriorly by the marginal branch of the cingulate cortex, posteriorly by the parietal-occipital fissure, and inferiorly by the subparietal sulcus [1]. Anatomical and connectivity studies have indicated that the precuneus belongs to a widespread network of cortical and subcortical structures [2]. Primary connections of the precuneus include the posterior cingulate and retrosplenial cortices, other regions of the parietal cortex, the frontal cortex, and the temporal-parietal-occipital area [1]. Among these connections, we focus on the frontoparietal circuits, as they are regarded as the main elements of the cortical motor system [3]. Each motor area receives afferents from a specific set of parietal territories. Studies have shown that area 5ci [4], which is in the anterior medial region of the precuneus, is essentially the secondary somatosensory area due to its close association with the supplementary motor area (SMA), and that areas 5 L and 5 M are connected to the superior parietal lobule (SPL), medial primary somatosensory area (SI), and motor cortex. The central region of the precuneus exhibits overlap with the mesial 7A and is connected to the dorsolateral and dorsomedial prefrontal cortices [5, 6].

Seizures arising from the precuneus are rare, and few studies have aimed to characterize the clinical presentation of such seizures within the anatomic context of the frontoparietal circuits. Previous studies are limited in that their analyses focused on parietal lobe epilepsy as a general entity [7]. These studies showed that an important feature of parietal lobe epilepsy is the polymorphism of ictal manifestation [8]. Parietal lobe epilepsy is usually associated with various auras, including somatosensory impairments; disturbances of body image; vertiginous sensations; and visual, auditory, or aphasic auras [9, 10]. Focal motor clonic activity, tonic posturing, and oral-gestural automatisms occur in 57, 28, and 17% of patients, respectively [11]. More recent studies have investigated seizures arising from subregions of the parietal lobe, including the precuneus. Seizures arising from the precuneus are more frequently associated with body movement sensations, visual auras, eye movements, vestibular manifestations, asymmetric tonic posturing, and hypermotor activity [1]. Precuneal epilepsy is difficult to differentiate from other types of epilepsy—particularly frontal lobe epilepsy—because seizure onset occurs at an anatomically deep and semiologically silent area [12]. As such seizures may spread to the frontal cortex, misinterpretation of the clinical manifestations can lead to incorrect localization. False localization has been reported in up to 16% of cases [13]. Hence, it is important to improve our understanding of the clinical and neurophysiological features of precuneal epilepsy to improve pre-operative evaluation and surgical strategies.

Stereoelectroencephalography (SEEG) offers distinct advantages over conventional noninvasive approaches, which may be insufficient due to the deep origin of precuneal epilepsy. First introduced by Talairach and Bancaud in the early 1960’s [14], SEEG is an invasive approach that can be used to reliably identify deeply buried anatomic structures, allowing one to construct a dynamic, three-dimensional (3D) spatiotemporal picture of epileptic activity [15]. Indeed, previous studies have utilized SEEG to precisely investigate the electrical activity of different brain structures involved in seizure generation and propagation [10, 16] .

In focal epilepsy, the symptoms are determined by the dynamic evolution of epileptic discharge from the initial seizure onset zone to the adjacent/distant cortical or subcortical areas. Therefore, characterizing the temporo-spatial evolution of seizure propagation and symptoms is crucial for improving our understanding of different clinical phenotypes [17, 18]. In the present study, we investigated the electroclinical characteristics of epilepsy originating from the precuneus, as well as the correlation between clinical phenotypes and the ictal pattern of seizure propagation.

## Methods

### Participants

The present retrospective study included 10 patients (9 men, 1 woman) with medically intractable epilepsy, all of whom underwent evaluation for surgical treatment at Yuquan Hospital and Xuan Wu Hospital between 2014 and 2016. The strategy for SEEG electrode implantation was decided upon during a multidisciplinary patient management conference at the Epilepsy Center. SEEG electrodes were implanted following comprehensive evaluation of each patient, which included a detailed history, video-EEG recording, MRI, PET, and other noninvasive localization methods. Patients with precuneal epilepsy were selected based on the following criteria: (a) SEEG-confirmed seizure onset from the precuneus; (b) extent of surgical excision limited or mostly limited within the precuneus; (c) post-operative follow-up at least every 6 months and ILAE Class 1–2 [19]; (d) no evidence of progressive brain disorders or systemic diseases. All included patients and caregivers provided written informed consent to participate and for publication. Patients in whom multiple epileptogenic zones or possible epileptogenic lesions were identified (e.g., tuberous sclerosis, multiple cavernous hemangioma) and those in whom independent epileptogenic cortical areas beyond the precuneus were confirmed using SEEG were excluded.

We began with 13 cases of electrodes embedded in the precuneus, but two of them were excluded due to diffuse electrodecremental events observed in the SEEG and uncertain seizure onset zones. An additional case was excluded due to bilateral epileptic foci and rejection of surgical treatment by the patient and family. Finally, we enrolled 10 patients. The clinical characteristics of the included patients are shown in Table 1. The mean age at seizure onset was 6.15 ± 6.2 years. The mean age at consultation was 13.6 ± 5.6 years. Our analyses focused on clinical semiology, as well as scalp EEG/SEEG findings.

**Table 1:** Demographics and clinical features of patients (*n* = 10)

Patient	Age/Sex **(**M/F**)**	Age at onset **(**Y**)**	MRI	PET hypometabolism	Hypothesis	Surgery	Pathology	ILAE class/Outcome (M)	Complication
1	17 M	8	Normal	Rt. F, rt. P	Rt. P, rt. F	Rt. PrC	FCD	1 (29)	None
2	6 M	2.5	Lt. P PG	Lt. F, lt. P	Lt. P, lt. F, lt. T	Lt. PrC, lt. SPL	PG	1 (23)	None
3	19 M	7.5	Normal	Rt. P	Rt. P, rt. F, rt. T	Rt. PrC, rt. PCC	FCD	2 (30)	None
4	20 M	16	Lt. P, lt. O	Lt. P	Lt. P, lt. O, lt. T	Lt. PrC, lt. Cu	EM	1 (35)	Rt. Inf. quadranopsia
5	8 M	3	Normal	Lt. T, lt. P	Rt. P, rt. T	Rt. PrC, rt. RSC, rt. PCC	FCD	2 (34)	None
6	17 M	3	Normal	Lt. T	Lt. P, lt. O, lt. T	Lt. PrC, lt. Cu	FCD	2 (33)	Rt. Inf. quadranopsia
7	19 M	19	Lt. P lesion	Normal	Lt. P, lt. F	Lt. PrC, lt. PCL	EM	1 (26)	Transient Rt. LE paresthesia
8	4 M	0.5	Normal	Bil. T	Lt.P, lt. T, lt. O, lt, I	Lt. PrC, lt. PCC	FCD	1 (18)	Upper respiratory infection
9	15F	1.5	Bil. P and bil. O lesion	Lt. T	Lt. P, lt. O, lt. F	Lt. PrC, lt. PCC, lt. Cu	EM	2 (37)	Rt. Inf. quadranopsia
10	11 M	0.5	Lt. P and bil. O lesion	Lt. T, lt. P	Lt. P, It. O, lt. T	Lt. PrC, lt. PCC, lt. Cu, It.RSC	EM	2 (37)	Rt. Inf. quadranopsia

*Bil* bilateral, *Lt* left, *Rt* right, *Inf* inferior, *F* frontal, *T* temporal, *P* parietal, *O* occipital, *I* insula, *PrC* precuneus, *SPL* superior parietal lobule, *PCC* posterior cingulate cortex, *Cu* cuneus, *RSC* retrosplenial cortex, *PCL* paracentral lobule, *FCD* focal cortical dysplasia, *PG* pachygyria, *EM* encephalomalacia, *LE* lower extremity

### Analysis of long-term scalp EEG monitoring and clinical semiology

Clinical characteristics and the results of scalp EEG monitoring are shown in Table 2. Prolonged EEG data were acquired via video-EEG monitoring (Nihon Kohdon, Japan or Bio-logic, USA). All patients underwent at least three typical seizures while awake and during sleep. The recording parameters were as follows: sampling rate = 1024 Hz, low filter setting = 0.16 Hz, high filter setting =70 Hz (Nihon Kohdon, Japan) or sampling rate = 256 Hz, low filter setting = 0.16 Hz, and high filter setting = 70 Hz (Bio-logic, USA). Each EEG sample was analyzed and classified according to the following criteria: (a) lateralization: (i) left, (ii) right, or (iii) not applicable (NA, when bilateral, generalized, or non-lateralized); (b) site: (i) frontal, (ii) temporal, (iii) parietal, (iv) occipital, (v) vertex, or (vi) not applicable (NA, when generalized or non-localized).

**Table 2:** Features of scalp EEG and semiology in patients (*n* = 10)

No	Interictal		Ictal		Semiology sequence	Seizure number	Seizure duration/frequence
	Lateralization	Site	Lateralization	Site			
1	NA	Central-parietal	NA	NA	Left limb tonic→hypermotor	13	20–30s; 2–3/d
2	NA	Temporal-parietal-occipital	NA	NA	Aura(indescribable discomfort) → eyes right deviation→bilateral asymmetric tonic seizure	6	90s–150s; 1–2/m
3	NA	Temporal-parietal-occipital	Rt	Temporal-parietal-occipital	Aura(vestibular response) → left versive→bilateral asymmetric tonic seizure→GTCS	3	2-7 min; 2–3/m
4	NA	NA	Lt	Temporal-parietal-occipital	Aura(vestibular response) → bilateral asymmetric tonic seizure→right versive→GTCS	5	90s–110s; 1–2/m
5	NA	Temporal-parietal-occipital	NA	NA	Aura(body image disturbance) → dyleptics→rapid eyes blinking→ bilateral asymmetric tonic seizure	3	150 s-5 min; 1–2/w
6	NA	Temporal-parietal-occipital	NA	NA	Aura(blur vision of eyes) → right version→bilateral asymmetric tonic seizure	10	15S-1min; 3–4/d
7	Left	Parietal	NA	NA	Aura(body image disturbance) → hypermotor	12	30s; 3–4/d
8	NA	NA	NA	NA	Dialeptic→bilateral asymmetric tonic seizure	4	15–30s; 5–6/d
9	NA	Diffused	NA	NA	Aura(somatosensory aura) → bilateral asymmetric tonic seizure	5	1 min 2–3/w
10	NA	Diffused	NA	NA	Right version→hypermotor→GTCS	6	3 min 3–4/w

Three independent clinicians utilized Lüders’ semiological seizure classification to evaluate ictal semiology and determine the semiological evolution sequence [18, 20]. For patients who experienced auras, aura information was derived from their previous medical history if disturbance of consciousness or postictal amnesia occurred during monitoring.

### Implantation of electrodes and analysis of anatomic-electrical-clinical correlations based on SEEG findings

A T1-weighted MRI scan and magnetic resonance angiography data were integrated to construct three-dimensional images using Neurotech stereotactic software (Neurotech, China). SEEG electrode implantation was based on a preimplantation hypothesis regarding the possible location of the epileptogenic zone (EZ). A Leksell headstock was installed on the morning of surgery, following which MRI scans were obtained and combined with previous MRI results to calculate the target position of each electrode (*x*, *y*, and *z* axes; α angle; β angle). Based on the calculated target positions, the electrodes were implanted using the Leksell stereotactic system. Postoperative high-resolution computed tomography (CT) scans were obtained to verify the exact location of each contact and to screen for postoperative complications. FreeSurfer (http://www.surfer.nmr.mgh.harvard.edu) was used to generate three-dimensional (3D) surface images of the brain, in order to determine the relationships of electrode position, the corresponding cortex, and surface vessels. Postoperative CT images were then co-registered with preoperative MR images using 3D Slicer (http://www.slicer.org) to confirm the actual locations of the SEEG electrodes.

The number of implanted SEEG electrodes ranged from 8 to 16 (median: 11.5) per patient, and the locations of the SEEG electrodes varied among patients. Signals were recorded by 5–18 contacts per intracranial electrode. SEEG data were recorded at a sampling rate of 2000 Hz and band-pass filtered between 0.16 Hz and 600 Hz (Nihon Koden, Japan), or at a sampling rate of 256 Hz and band-pass filtered between 0.16 Hz and 100 Hz (Bio-logic, USA). SEEG electrodes were referenced to screw electrodes placed at the vertex or to white matter electrodes.

Ictal onset and propagation were retrospectively analyzed by three clinicians based on the SEEG data. Following implantation, the clinicians analyzed the semiological sequence, ictal SEEG evolution, and the underlying anatomical structures for each seizure. As the seizure evolved, we marked the time points corresponding to each symptom and the electrode contacts exhibiting fast SEEG rhythms, following which we determined the corresponding anatomical locations on MR images. Finally, we analyzed the relationship between the anatomical position of the fast rhythm and the appearance of symptoms based on seizure evolution, as well as the anatomical-electrical-clinical relationship of each seizure.

### Surgery and pathology

The surgical procedure was performed with controlled respiration under general inhalation anesthesia. The extent of surgical resection was based on a review of the interictal and ictal changes, extraoperative cortical stimulation functional map, and imaging data collected, which were used to reach consensus at a multidisciplinary patient management conference. Areas of the brain targeted for resection were removed using a microneurosurgical technique. During the surgery, the earlier video EEG findings and intraoperative ECoG information were used to help to further identify the area targeted for resection, which typically included the ictal onset area and adjacent areas where frequent interictal spikes were demonstrated. Surgical specimens were fixed in 10% buffered formalin, embedded in paraffin, and then stained.

## Results

### Semiological characteristics

#### Aura

Seven of the 10 included patients experienced auras. Two patients experienced body image disturbance (Pt. 4 and 7) (i.e., a feeling that an extremity has spatial displacement), two patients experienced vestibular responses (Pt. 3 and 5), and one patient experienced a somatosensory aura (Pt. 9) (i.e., numbness in the bottom of the right foot spreading upwards toward the knee). One patient experienced a visual aura (Pt. 6) (i.e., blurred vision in both eyes), while one patient experienced a non-specific aura characterized by discomfort in the heart (Pt. 2).

#### Motor manifestations

Seven of the 10 included patients experienced BATS (Pt. 2, 3, 4, 5, 6, 8, and 9), while the remaining three experienced HMS (Pt. 1, 7, and 10). Patient 1 exhibited horizontal movement of the trunk and hips accompanied by stiffness in the contralateral limbs. Patient 7 exhibited trunk and hip movement accompanied by pedaling motions in the bilateral lower extremities. Patient 10 exhibited right upper limb flapping accompanied by pedaling motions in the bilateral lower extremities and exaggerated facial expressions. In addition, four patients exhibited continuous and vigorous bilateral conjugated eye movements towards the side contralateral to the epileptic region, which were accompanied by synchronous head movements.

### Interictal and ictal EEG data

All patients underwent video-EEG monitoring. In Patient 7, interictal epileptiform discharge occurred ipsilateral to the side of the EZ. Discharge was non-lateralized in eight patients, and one patient (Pt. 4) exhibited no interictal discharge. In six patients, interictal EEG signals were mainly localized to the temporal-parietal-occipital area. In the remaining four patients, interictal discharge was generalized or non-localized.

In two patients (Pt. 3, 4), the ictal onset occurred ipsilateral to the EZ, and the site of seizure onset was primarily localized in the temporal-parietal-occipital area. In the remaining patients, ictal EEG signals were generalized or non-localized.

### Anatomic-electrical-clinical correlations based on SEEG findings

**Fig. 1:**
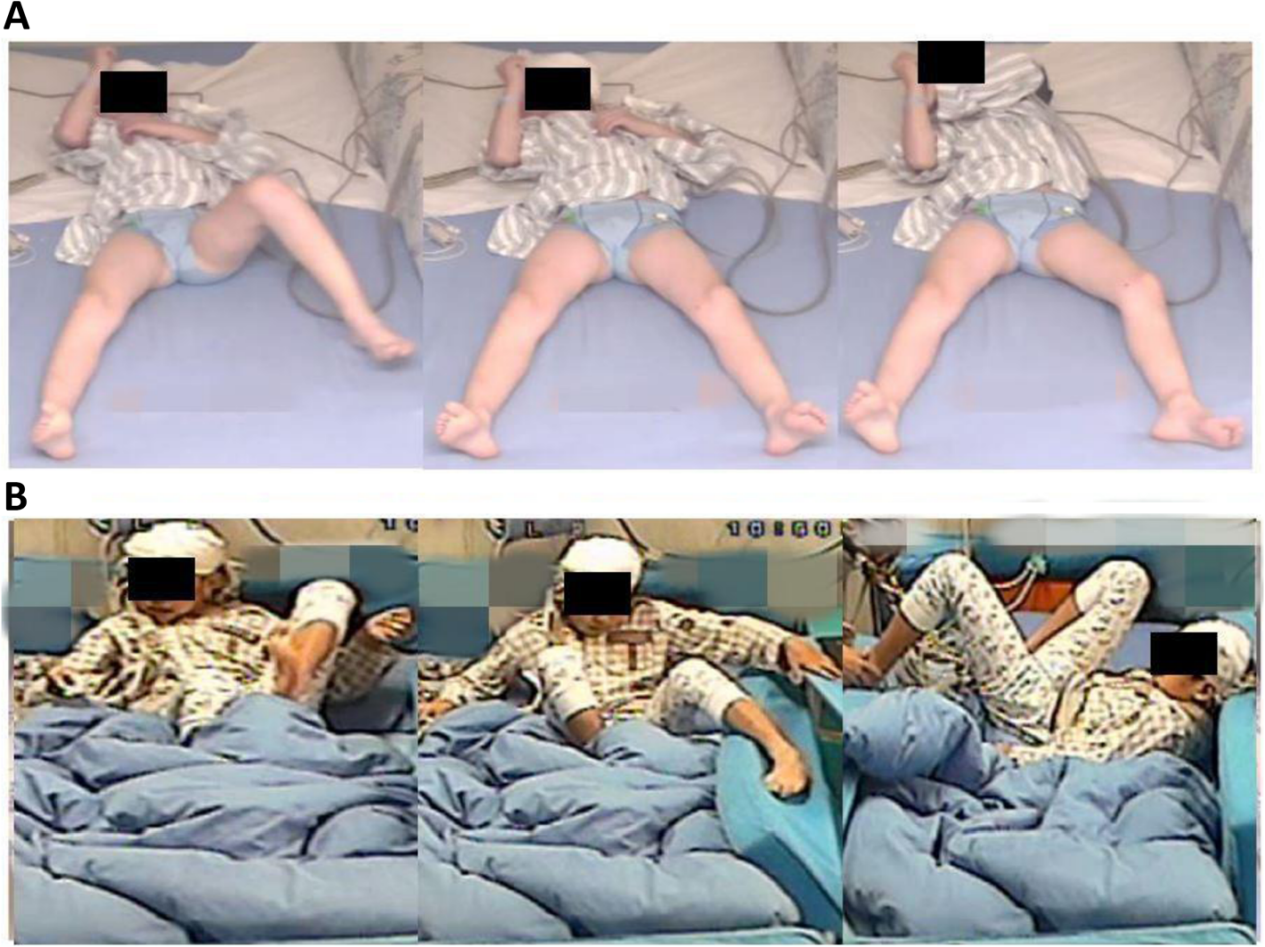
Seizure samples. Video samples of the BATS of patient 2 (**a**) and the HMS of patient 10 (**b**). Signed consent forms authorizing publication have been obtained for all identifiable patients

Two patterns of anatomic–electrical–clinical correlations were observed. The first pattern was characterized by BATS activity with ictal spread to the supplementary motor area (SMA), paracentral lobule (PCL), postcentral gyrus (PoCG), or precentral gyrus (PrCG) of the ipsilateral hemisphere. Seven of the ten included patients exhibited BATS (Fig. 1a). However, due to limitations in the number and scope of the electrodes, data from only three patients were used to explore the frontoparietal circuits.

**Fig. 2:**
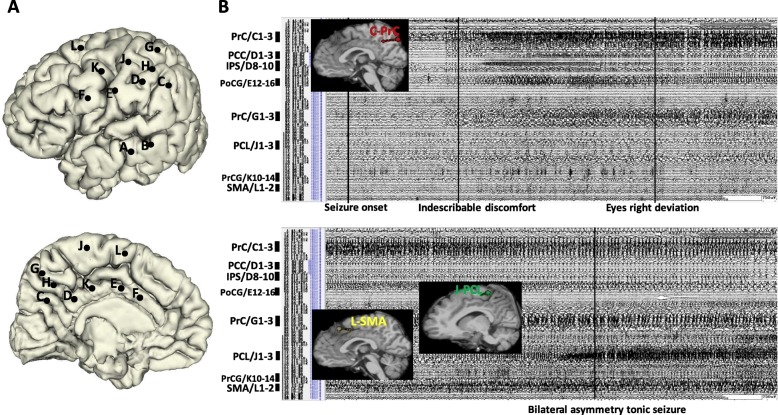
**a** SEEG electrode implantation on lateral and medial view of three-dimensional (3D) brain of Patient 2. **b** Ictal SEEG trace and two-dimension (2D) T1-weighted sagittal images with 3D electrodes showing the key target points. It shows that the seizure onset was recorded from precuneus (PrC) (medial contacts of electrode C1–3) with no clinical sign being observed. When the ictal activity spread to PCL (medial contacts of electrode J1–3), PrCG (lateral contacts of electrode K10–14), and SMA (medial contacts of electrode L1–2), the clinical sign of bilateral asymmetry tonic seizure appeared

The ictal SEEG trace of Patient 2 revealed that seizure onset was characterized by the appearance of fast, low-voltage discharge (gamma band) in the precuneus (mesial contacts of electrode C1–3). After 5 s, an indescribable aura appeared. Approximately 20 s later, seizure activity spread to the PCC (mesial contacts of electrode D1–3), intraparietal sulcus (IPS) (middle contacts of electrode D8–10), and PoCG (lateral contacts of electrode E12–16). At this time, rightward deviation of the eyes was observed. A BATS was observed only when ictal activity had propagated to the PCL (mesial contacts of electrode J1–3), PrCG (lateral contacts of electrode K10–14), and SMA (mesial contacts of electrode L1–2) (Fig. 2).

**Fig. 3:**
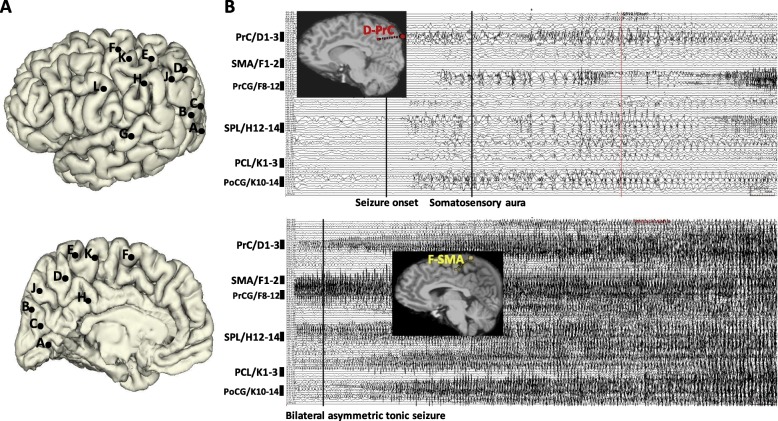
**a** SEEG electrode implantation on lateral and medial view 3D brain of Patient 9. **b** Ictal SEEG trace and 2D T1-weighted sagittal images with 3D electrodes showing the key target points. It shows that the seizure onset was recorded from PrC (medial contacts of electrode D1–3) with no clinical sign being observed. When the seizure spreads to SMA (medial contacts of electrode F1–2), PrCG (lateral contacts of electrode F8–12), PCL (medial contacts of electrode K1–3), and PoCG (lateral contacts of electrode K10–14), the semiology of bilateral asymmetry tonic seizure appeared

The ictal SEEG recording of Patient 9 revealed that seizure onset occurred in the precuneus (mesial contacts of electrode D1–3). Two seconds later, a somatosensory aura was observed. BATS activity appeared once the seizure had spread to the SMA (mesial contacts of electrode F1–2), PrCG (lateral contacts of electrode F8–12), PCL (mesial contacts of electrode K1–3), and PoCG (lateral contacts of electrode K10–14) (Fig. 3).

**Fig. 4:**
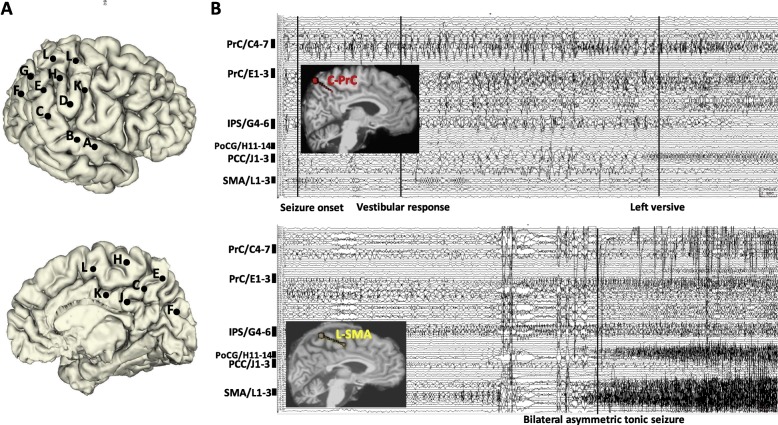
**a** SEEG electrode implantation on lateral and medial view of 3D brain of Patient 3. **b** Ictal SEEG trace and 2D T1-weighted sagittal images with 3D electrodes showing the key target points. It shows that the seizure onset was recorded from a relatively focal region of PrC (middle contacts of electrode C4–7 and mesial contacts of electrode E1–3). It is only when the seizure spreads to PoCG (lateral contacts of electrode H11–14) and SMA (medial contacts of electrode L1–3), that the semiology of BATS appears

The ictal SEEG recording of Patient 3 revealed that seizure onset occurred within a relatively focal region of the precuneus (middle contacts of electrode C4–7 and mesial contacts of electrode E1–3). Ten seconds later, seizure activity had spread to the PCC (mesial contacts of electrode J1–3) and IPS (middle contacts of electrode G4–6). A BATS was observed only once seizure activity had spread to the PoCG (lateral contacts of electrode H11–14) and SMA (medial contacts of electrode L1–3) (Fig. 4).

**Fig. 5:**
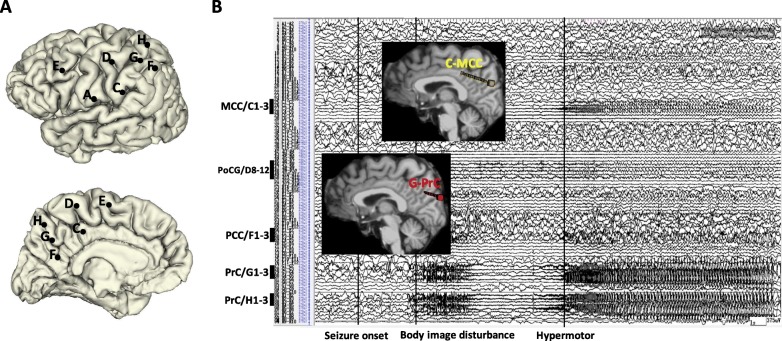
**a** SEEG electrode implantation on lateral and medial view of 3D brain of Patient 7. **b** Ictal SEEG trace and 2D T1-weighted sagittal images with 3D electrodes showing the key target points. It showed that seizure onset was recorded from PrC (medial contacts of electrode G1–3 and electrode H1–3) with no clinical sign being observed. When the seizure spreads to MCC (medial contacts of electrode C1–3), PCC (medial contacts of electrode F1–3), and PoCG (lateral contacts of electrode D8–12), the semiology of hypermotor appeared

The second pattern was characterized by HMS activity with ictal spread to the PCC and MCC of the ipsilateral hemisphere, which was observed in three patients (Fig. 1b). However, due to electrode limitations, data from only two patients were used to investigate the frontoparietal circuits. The ictal SEEG trace of Patient 7 revealed that seizure onset occurred within the precuneus (medial contacts of electrode G1–3 and H1–3), in the absence of clinical signs. Hypermotor signs were observed once seizure activity had spread to the MCC (medial contacts of electrode C1–3), PCC (medial contacts of electrode F1–3), and PoCG (lateral contacts of electrode D8–12) (Fig. 5).

**Fig. 6:**
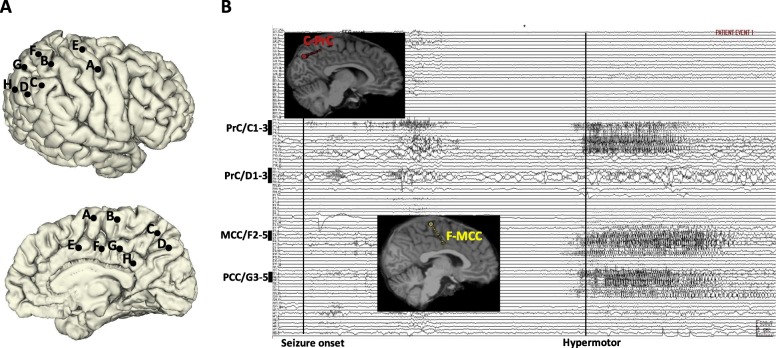
**a** SEEG electrode implantation on lateral and medial view of 3D brain of Patient 3. **b** Ictal SEEG trace and 2D T1-weighted sagittal images with 3D electrodes showing the key target points. It shows that seizure onset was recorded from PrC (medial contacts of electrode C1–3 and electrode D1–3). Only when the seizure spread to MCC (medial contacts of electrode F2–5) and PCC (medial contacts of electrode G3–5), the semiology of hypermotor emerged

The ictal SEEG trace of Patient 1 revealed that seizure onset was characterized by a transition from preictal rhythmic spiking to fast discharge (gamma band) in the precuneus (medial contacts of electrode C1–3 and electrode D1–3), which occurred over the course of approximately 6 s, in the absence of clinical signs. Hypermotor signs were observed only once seizure activity had spread to the MCC (medial contacts of electrode F2–5) and PCC (medial contacts of electrode G3–5) (Fig. 6).

### Surgical complications

Surgical complications were observed in six patients. Four (Pt. 4, 6, 9, and 10) showed right inferior quadranopsia following resection, which was considered a complication of resection of the precuneus and cuneus. One (Pt. 7) had a transient contralateral lower extremity paresthesia, which was considered a result of postoperative edema causing transient damage to the paracentral lobule, and the symptom resolved 2 weeks later. One (Pt. 8) had upper respiratory infection on the third day after operation. This may be related to the patient’s young age and weak resistance; the patient returned to normal after appropriate anti-inflammatory treatment.

## Discussion

In the present study, we analyzed semiological characteristics and EEG/SEEG data in order to identify anatomic–electrical–clinical relationships in 10 patients with precuneal epilepsy. Previous studies have revealed that precuneal epilepsy is associated with several types of premonitory auras [1]. In one such study, visual auras were reported in three patients (50%), vestibular responses in two patients (33%), and sensations of falling or movement in one patient [10]. Previous studies have demonstrated that stimulation of the anterior portion of the precuneus results in body image disturbance, while stimulation of the posterior portion results in visuomotor illusions [21, 22]. Additional studies have reported that stimulation of the precuneus evokes vestibular responses [23]. Our findings are mostly consistent with these previous studies, although body image disturbance occurred more frequently in our patient group, perhaps because most patients exhibited seizure activity in the anterior precuneus.

We observed two major patterns of activity in patients of the present study: BATS and HMS. BATS is often associated with seizure activity in the mesial parietal region [24], similar to patterns observed in patients with mesial frontal lobe epilepsy. However, seizures originating from the precuneus are typically briefer than those with an SMA onset, and patients with precuneal seizures are typically quicker to regain awareness [25]. These features were also observed in our patients who experienced BATS.

Some studies have reported that seizures originating from the lateral and mesial parietal regions may manifest as hypermotor activity [26]. Such hypermotor activity was also observed in some patients of the present study. However, HMS typically has a frontal lobe onset and occur in 15–27% of patients with frontal lobe epilepsy, in which seizures primarily rise from the orbitofrontal or mesial frontal cortex [27–29]. In such cases, it is difficult to distinguish seizures arising from the parietal lobe from those arising from the frontal lobe. However, some studies have indicated that the type of aura and the delay between seizure onset and hypermotor activity can be used to make this distinction [26]. As previously mentioned, precuneal HMS is often preceded by unique auras (e.g., body image disturbance and vestibular responses). In addition, patients with frontal lobe seizures almost always experience hypermotor activity at seizure onset or during the first third of the seizure [30]. However, in patients with precuneal seizures, there is usually a substantial delay between seizure onset and the appearance of hypermotor activity, which may represent the time required for the seizure to propagate from the precuneus to the frontal lobe or to subcortical structures [31, 32].

In precuneal epilepsy, the interictal EEG provides little clue to the lateralization and localization of seizure activity [9], as discharge is prone to spread to other cerebral lobes and induce an error in localization. The widespread connectivity between the precuneus and other cerebral regions may represent the underlying cause of errors in localization. Hence, it is difficult to distinguish precuneal from non-precuneal seizures via scalp EEG, particularly those arising from the mesial frontal lobe. SEEG represents a unique method for identifying and characterizing the underlying physiologic mechanisms of precuneal seizures.

In SEEG methodology, the EZ is defined as the site of origin for epileptic seizures [33]. Therefore, we focused our SEEG analyses mainly on the identification of the brain regions in which specific ictal patterns developed during seizures [34, 35]. The region associated with the primary organization of ictal discharge and subsequent propagation is correlated with the anatomical positions of the electrodes that display maximal abnormal activity, and with the evolution of clinical seizure signs. At present, however, the identification of the EZ is based on the ability of experienced clinical neurophysiologists to identify the relevant anatomic–electrical–clinical correlations and SEEG patterns.

In the present study, we analyzed the ictal SEEG of five patients in whom the relevant electrodes involved the frontoparietal network. In three cases, BATS was observed only once the ictal activity had spread to the SMA, PCL, PrCG, or PoCG. Our results indicated that abnormal ictal activity is easily propagated from the precuneus to these regions, suggesting that the precuneus is functionally connected to the SMA and other central areas. Some previous studies have reported that the medial aspect of the posterior parietal lobe is connected to the SMA and premotor areas [4, 36]. Therefore, seizures occurring within the medial side of the parietal lobe may spread to the SMA, causing bilateral asymmetric tonic manifestations [24].

In two patients of the present study, HMS was observed only when ictal activity had spread to the PCC and MCC. Some studies have reported that the middle cingulate gyrus receives afferent connections from the medial surface of the parietal lobe, particularly the precuneus. Such connections provide information regarding noxious stimulation in the body, directing the motor area of the cingulate gyrus to initiate evasive actions/behaviors. Thus, seizure activity originating from the precuneus may induce hypermotor movements [37]. In one case series, two patients with electrodes implanted in the motor aspect of the MCC exhibited fast discharge in this region during hypermotor movements [26]. Thus, hypermotor activity associated with precuneal seizures may be due to the ease with which abnormal ictal activity propagates from the precuneus to the PCC and MCC. Taken together, the accumulated evidence suggests that the precuneus is functionally connected with the PCC and MCC.

The present study possesses several limitations of note. First, the study was limited by the number and coverage of the implanted electrodes. The spatial limitations of recording may have caused difficulties in identifying all possible pathways of seizure propagation. The study was also limited by the small number of included cases and its retrospective design. Nonetheless, our study provides valuable evidence regarding the electroclinical characteristics of precuneal epilepsy, as well as the possible mechanisms underlying variations in semiology based on SEEG findings within frontoparietal circuits. Such findings may represent clinical indicators of precuneal epilepsy. Future studies should investigate further subdivisions of the precuneus using a greater number of electrodes in order to provide a more complete characterization of precuneal epilepsy.

## Conclusion

The clinical characteristics of precuneal epilepsy are complex and various. Aura type (e.g., body image disturbance and vestibular response), BATS, and HMS are the main indicators of precuneal epilepsy. Scalp EEG is of little use when attempting to localize precuneal seizures. Analysis of high-quality SEEG data can help to identify the relevant anatomic-electrical-clinical correlations. Our findings indicate that the clinical characteristics of precuneal epilepsy are different among patients, and that the final electro–clinical phenotype depends on the pattern of seizure spread.

## Abbreviations

2D: Two-dimensional
3D: Three-dimensional
BATS: Bilateral asymmetric tonic seizure
CT: Computed tomography
EEG: Electroencephalography
EZ: Epileptogenic zone
HMS: Hypermotor seizure
IPS: Intraparietal sulcus
MCC: Middle cingulate cortex
MRI: Magnetic resonance imaging
PCC: Posterior cingulate cortex
PCL: Paracentral lobule
PET: Positron emission tomography
PoCG: Postcentral gyrus
PrC: Precuneus
PrCG: Precentral gyrus
SEEG: Stereoeletroencephalography
SMA: Supplementary motor area
SPL: Superior parietal lobule
